# Paget-Schroetter Syndrome (PSS) and Adjunctive Treatment With Mechanical Aspiration Thrombectomy and Catheter Directed Thrombolysis: A Case Report and Review of Literature

**DOI:** 10.7759/cureus.24437

**Published:** 2022-04-24

**Authors:** Amitoj Singh, Mehrvaan Kaur, Amroz Singh, Ammar Bhatti, Rushin Patel

**Affiliations:** 1 General Cardiology, Lahey Hospital and Medical Center, Burlington, USA; 2 Department of Diagnostic Radiology, St. Joseph Mercy Oakland Hospital, Pontiac, USA; 3 Internal Medicine, Dayanand Medical College and Hospital, Ludhiana, IND; 4 Internal Medicine, Lahey Hospital and Medical Center, Burlington, USA

**Keywords:** pvd: peripheral vascular disease, paget-schroetter syndrome, deep vein thrombosis (dvt), mechanical thrombectomy (mt), catheter-directed thrombolysis

## Abstract

Paget-Schroetter syndrome, also known as venous thoracic outlet syndrome, is primarily an effort-induced thrombosis of the subclavian and axillary veins. Treatment modalities involve systemic anticoagulation, catheter-directed thrombolysis (CDT), and surgical decompression. Early endovascular intervention is noted to improve outcomes and result in symptomatic relief. Here we implore the usage of the novel mechanical aspiration thrombectomy device as an adjunct to CDT for the management of peripheral venous thrombosis and highlight it as a treatment option resulting in substantial radiological and symptomatic improvement.

## Introduction

Thoracic outlet syndrome (TOS) refers to a group of disorders that presents with features of neurovascular bundle compression as it traverses through the thoracic outlet. According to the etiology, it is divided into neurogenic, venous, or arterial TOS. Neurogenic comprises 95% of all cases, with venous TOS encompassing 4%, followed by arterial with less than 1% contribution [1]. Venous TOS, also referred to by the eponym Paget-Schroetter syndrome, is primarily an effort-induced thrombosis of the subclavian and axillary veins. This syndrome results from excessive irritation or impingement of the primary vein owing to repetitive straining of the involved extremity. Populations with an already narrow costoclavicular space, through which the vein traverses, are highly predisposed. It is usually seen in the young athletic population with or without an underlying coagulopathy, who present with arm swelling, pain, and engorgement of veins in the chest wall. Diagnosis is made with imaging modalities, including duplex ultrasonography and computed tomographic (CT)/Magnetic resonance (MR) venography. Standard treatment measures include anticoagulation and thrombolytic drugs, with more advanced approaches utilizing catheter-directed thrombolysis (CDT) and/or pharmacomechanical thrombectomy (PMT), with surgical decompression once the initial blood clot is dissolved. This case highlights the use of mechanical aspiration thrombectomy (penumbra device) as an adjunctive treatment to CDT for the management of venous TOS resulting in early symptomatic relief and substantial radiographic improvement.

## Case presentation

**Figure 1:**
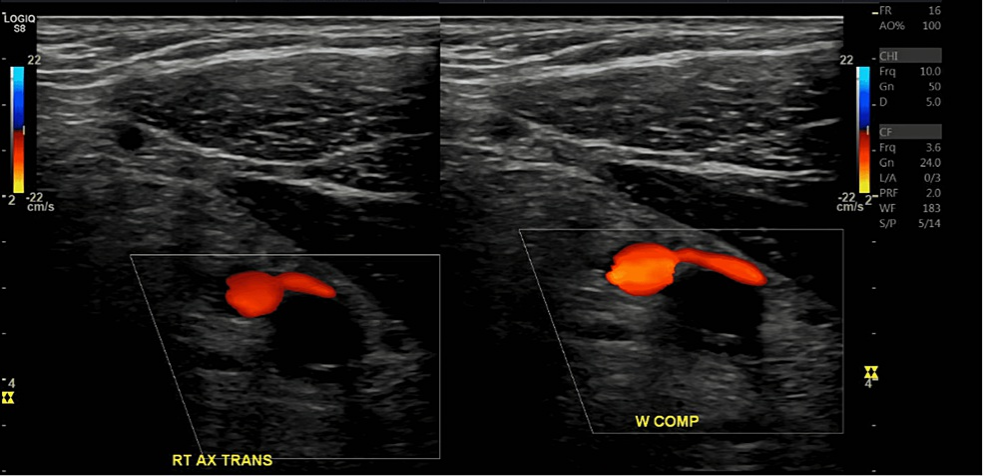
Duplex ultrasound reveals non-compressibility and lack of flow in the right axillary vein consistent with occlusive deep vein thrombosis

**Figure 2:**
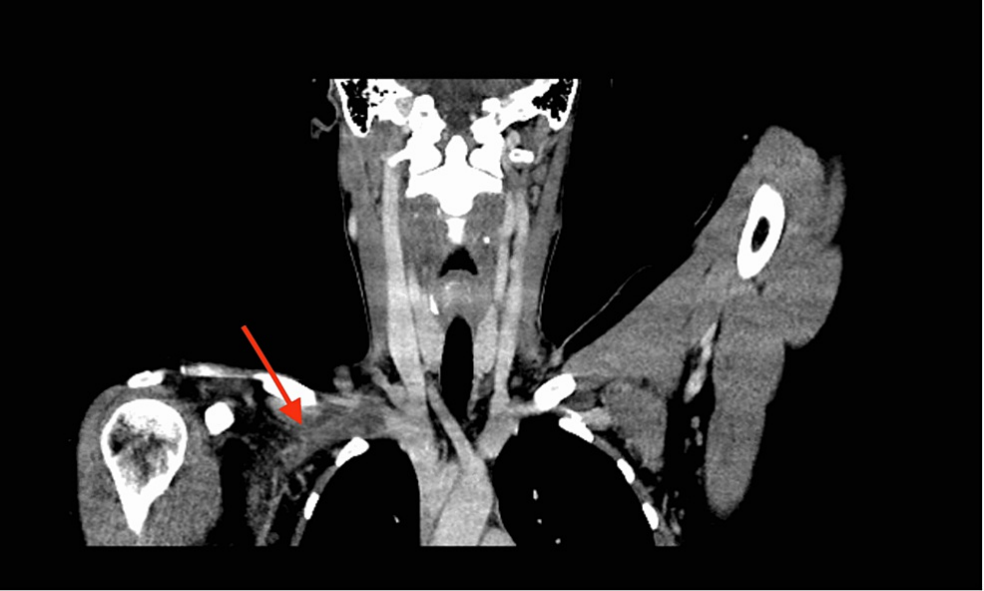
Delayed phase on CT angiography demonstrating a large filling defect in the mid and distal right subclavian vein. The right internal jugular and superior vena cava were patents

A 24-year-old female on oral contraceptives (OCPs) presented to the emergency department with three days of right upper extremity swelling and discomfort with movement. She leads an active lifestyle, including regular workout and practices axe throwing. Right upper extremity physical examination demonstrated visible swelling, tenderness to palpation over the biceps, and prominent venous vasculature. Pertinent history was confirmed with the patient, including no prior history of thrombosis or contributing family history. Laboratory studies revealed hemoglobin of 11.7g/dL, platelet count of 133,000/uL, and an international normalized ratio (INR) of 1.0. Beta-HCG testing was negative for pregnancy. Vascular ultrasound demonstrated deep venous thrombosis (DVT) within the right subclavian and axillary veins (Figure 1). She underwent computed tomography angiography (CTA) of the chest, which was negative for pulmonary embolus (PE); however, redemonstrated acute DVT without extension into the superior vena cava (SVC) or the right jugular vein. No regional masses were identified (Figure 2). A diagnosis of acute DVT of the right axillary and subclavian veins attributed to effort-induced thrombosis was made. Oral anticoagulation with Rivaroxaban 15mg twice a day was prescribed, and OCPs were discontinued. Despite six days of treatment, she remained symptomatic with tenderness and swelling of the right upper extremity.

**Figure 3:**
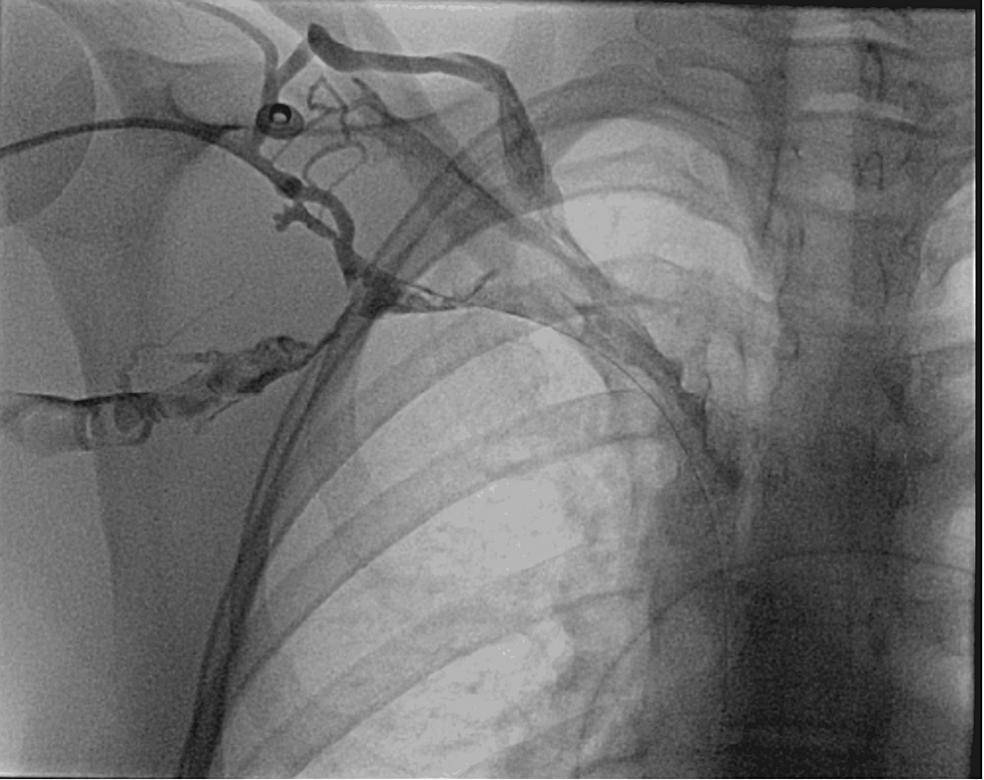
Axillary venography demonstrating complete occlusion of the right subclavian vein

The patient was taken to the catheterization lab and underwent venography through the right forearm, demonstrating total occlusion of the subclavian vein (Figure 3), which was partially recanalized with balloon angioplasty; however, with significant clot burden was visualized in the axillary vein.

**Figure 4:**
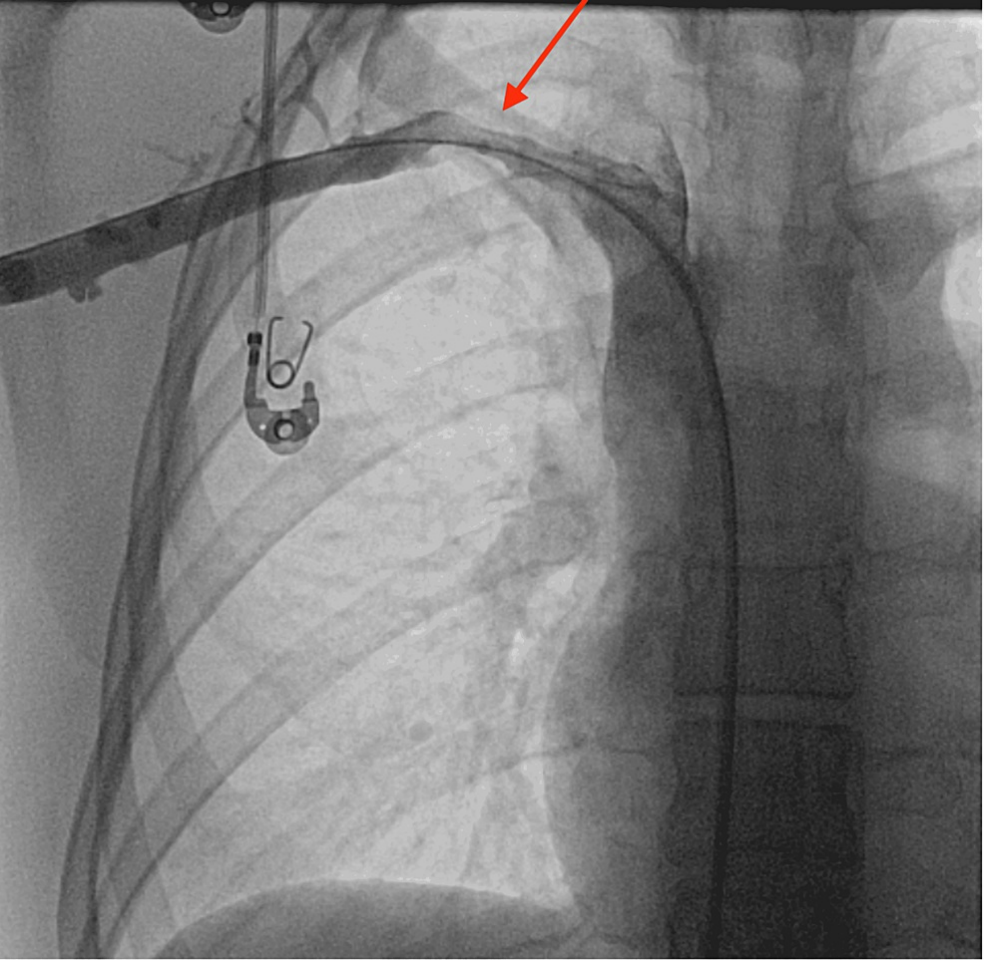
Post catheter directed thrombolysis showing recanalization of the subclavian and axillary veins but with residual thrombus in the mid subclavian vein at the site of compression

The patient was noted to have severe venospasm unremitting to increasing sedation and nitroglycerin instillation. This hindered the placement of the penumbra catheter leading to the subsequent deployment of an infusion catheter for the instillation of tissue plasminogen activator (tPA), starting at a rate of 1mg/hr. Heparin drip was started at the rate of 0-42 units/kg/hr, and the patient was admitted to the cardiac care unit for overnight management. Anti-factor-Xa levels were followed post heparin initiation, which was within the therapeutic range (0.1-0.3 IU/mL). After 12hrs of tPA infusion, repeat venography was performed. An attempt to reaccess the right basilic vein was unsuccessful due to severe venospasm. Therefore, the right common femoral vein access was obtained with an 18 G needle using the modified Seldinger technique, and an 8 Fr Cordis 11 cm sheaths (Cardinal Health, USA) was placed. Venography demonstrated significant recanalization of the axillary and subclavian veins but with persistent moderate thrombus burden (Figure 4) at the site of compression.

**Figure 5:**
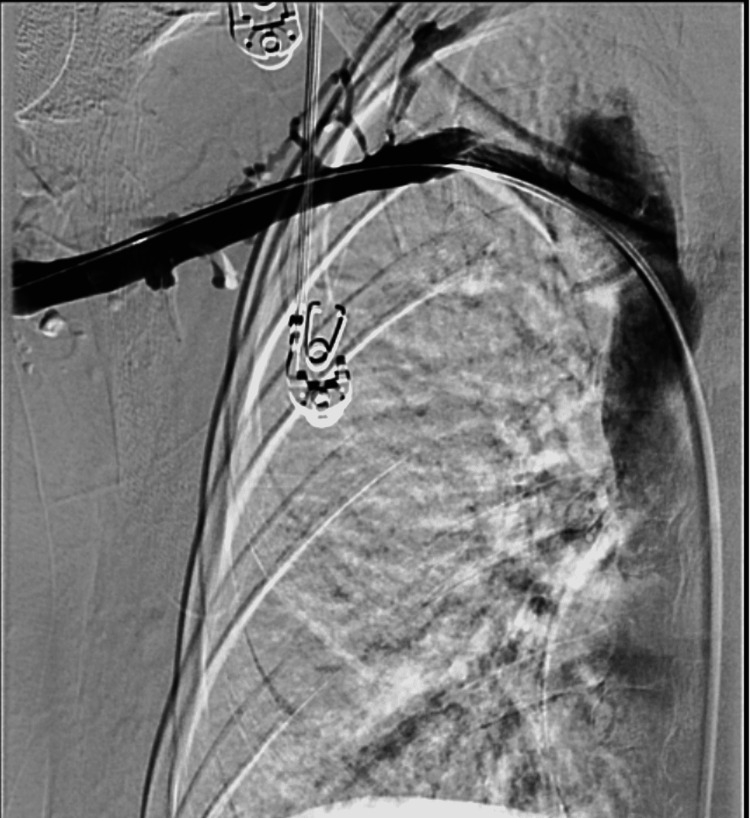
Digital subtraction venography post mechanical thrombectomy with significant improvement in the thrombus burden

Given this thrombus burden, we performed mechanical aspiration thrombectomy with the penumbra device leading to substantial improvement in flow and successful recanalization with 30% residual stenosis (Figure 5).

Subsequently, balloon angioplasty using 6.0 x 40 mm balloon was performed. There were no immediate complications. The patient reported marked improvement in symptoms the following day, and she was discharged home on Rivaroxaban for three months. She was referred to cardiothoracic surgery to explore potential surgical therapies for PSS. The patient was educated regarding pregnancy planning and post-thrombotic syndrome. She ultimately underwent surgery for thoracic outlet syndrome, including first rib resection and C7 scalenectomy, and continued to be symptom-free on follow-up visits.

## Discussion

A myriad of approaches have been described in the literature for treatment of acute DVT. However, there is no clear consensus on the best initial approach; hence treatment should be tailored to the underlying causative factors and reviewed on a case-to-case basis, based on the clinical presentation, severity and resource availability.

The cornerstone of the treatment is anticoagulation which can vary from utilizing subcutaneous low molecular weight heparin (LMWH) to intravenous unfractionated heparin with monitoring to the newer target-specific oral anticoagulants. Anticoagulation by itself prevents thrombus propagation and its embolization but is limited by the fact that it does not lyse the existent thrombus and does not prevent post-thrombotic complications. The amelioration of symptoms following anticoagulation is related more to the development of collateral veins rather than the resolution of the thrombus itself [2].

The second option to the treatment strategy includes thrombolysis for better resolution of the clot burden leading to symptomatic benefit. Catheter directed thrombolysis (CDT) is preferred over systemic thrombolysis to avoid excessive exposure to tissue plasminogen activator (tPA) and potentially limit the adverse bleeding risks. It is noteworthy that CDT is indicated for patients with an acute clot of less than 14 days duration and without any contraindications to thrombolysis. A clot of more than two weeks in duration is associated with limited success attributed to thrombus organization [3]. Hence, if the patient is symptomatic, presents in a timely fashion, or has a massive clot burden, the use of thrombolytic therapy is advocated. 

However, the use of CDT is not entirely devoid of risks. When compared with anticoagulation alone, the use of CDT results in significant complications, including risks of major bleeding and PE [4]. The higher risk of bleeding is explained by the fact that using CDT exposes patients to a longer duration and higher cumulative dosage of tPA. Moreover, administration of CDT therapy requires monitoring in the intensive care unit for the duration of treatment leading to higher procedural costs and healthcare resource utilization. 

To mitigate time and costs, there have been limited studies and case reports utilizing pharmacomechanical thrombectomy (PMT) as a therapeutic approach for the removal of thrombus. Usage of PMT results in improved restoration of patency and low severity of post thrombotic sequelae when compared to CDT therapy alone [5]. Apart from the time to achieve lysis, which was significantly shorter with PMT in comparison to CDT, it also resulted in being more cost effective, with one study documenting a reported savings of 1776$ when compared to the overall cost of CDT therapy [6]. When used in combination with CDT, it brings down the exposure time to tPA, thereby reducing the bleeding complications [7]. It also reduces the length of stay required in intensive care settings and the overall hospital cost.

However, it is worthwhile to remember that in cases of Paget-Schroetter syndrome, the usage of thrombolysis and anticoagulation results in symptomatic success, but there is usually an underlying factor resulting in the extrinsic compression that needs to be addressed using surgical approach to prevent recurrent episodes.

## Conclusions

Paget-Schroetter syndrome is an effort induced thrombosis of the subclavian and axillary veins. The fundamental approach to treatment is anticoagulation, with more advanced strategies, including catheter directed thrombolysis and pharmacomechanical thrombectomy. Mechanical thrombectomy (Penumbra device) can be used as primary or adjunctive therapy along with catheter directed thrombolysis for treatment. The Penumbra device is a novel system that generates a near vacuum enabling removal of thrombus from vessels of the peripheral venous and arterial system resulting in significant improvement within the patency of the vascular lumen and restoration of normal flow. The combination results in better safety profile for the patient, early symptomatic relief and lesser stay in intensive care settings. However, identifying the underlying cause of Paget-Schroetter syndrome is paramount to preventing recurrent episodes.
